# Relationship between Vitamin D Status and Striae Distensae: A Case-Referent Study

**DOI:** 10.1155/2015/640482

**Published:** 2015-11-09

**Authors:** Rafaela Koehler Zanella, Denis Souto Valente, Leo Francisco Doncatto, Daniele Dos Santos Rossi, Aline Grimaldi Lerias, Alexandre Vontobel Padoin

**Affiliations:** ^1^Mãe de Deus Health System, Rua Soledade 569, 90470-340 Porto Alegre, RS, Brazil; ^2^Graduate Program in Medicine and Health Sciences, School of Medicine, PUCRS (FAMED), Avenida Ipiranga 6681, 90619-900 Porto Alegre, RS, Brazil; ^3^ULBRA School of Medicine, Lutheran University of Brazil (ULBRA), Avenida Farroupilha 8001, 92425-900 Canoas, RS, Brazil; ^4^School of Medicine, PUCRS (FAMED), Avenida Ipiranga 6681, 90619-900 Porto Alegre, RS, Brazil

## Abstract

Vitamin D (VD) plays a role in the skin regulation. Striae Distensae (SD) are manifestations of epidermal atrophy that occurs after tissue tearing due to overstretching or rapid growth. The objective of this study was to investigate the relation between serum VD and occurrence of SD in women who had undergone mammaplasty with silicone implants. A case-referent study was conducted. The blood values of 25-hydroxyvitamin D (25OHD) were measured before the surgery. For each patient postoperatively diagnosed with SD, four other participants submitted to the same surgery, without the development of SD, were enrolled as the healthy controls. 67 women with SD after the surgery entered the study. 268 formed the control group. In the serum of healthy controls 25OHD mean was 27 ng/mL, and SD cases presented 20 ng/mL (*P* = 0.01). Scarce values of VD have been observed in 56.71% of the cases presenting SD and in 39.91% without SD (*P* = 0.002). Chance of having VD values lower than 20 ng/mL amongst cases with SD is 2.38 (*P* = 0.0001). Lower serum levels of VD are linked to a higher occurrence of SD.

## 1. Introduction

It is nowadays proven that the physiological significance of vitamin D (VD) status spreads far beyond the bone metabolism regulation [1]. Lately, a huge rise in the number of clinical studies explaining the VD decisive role in a plenty of physiological functions and associating lower levels of VD with lots of diseases has been observed. Deficit of VD is currently recognized as a worldwide pandemic [2, 3]. VD deficit major cause is the absence of appraisement that the major source of VD in all ages is sun exposure. Throughout life, VD plays a decisive role in the maintenance and development of a healthy body. A controversy still remains in relation to the serum level of 25-hydroxyvitamin D (25OHD) that must be attained for both bone health and reducing risk for VD deficit associated illness and how much VD must be supplemented [4, 5]. Having normal VD in the skin helps reduce acne, boost cutaneous elasticity, stimulate the dermal collagen production, and probably reduce skin cancer incidence [6–8].

Striae Distensae (SD), also known as stretch marks, are manifestations of an epidermal atrophy occurring after tissue tearing due to overstretching or rapid growth and are characterized by distinct microstructural features. Atrophic, linear, and parallel lesions usually characterize SD. The lesions are usually running perpendicular to Langer's lines, which represent the direction of minimum extensibility [9, 10]. The literature review performed using PUBMED and SciELO finds a 7.06% to 4.60% frequency of occurrence of SD following augmentation mammoplasty [11, 12].

The main objective of the present study has been to investigate the relation between serum VD and occurrence of SD in women who had undergone breast augmentation surgery with silicone implants.

## 2. Patients and Methods

A case-referent study has been conducted and the data of individuals who underwent breast augmentation surgery with silicone implants in a private clinic was analyzed. Data has been collected from patients' electronic records stored in RMD Clinic Software (RDTI Systems, Brazil) starting from January 2005 to March 2015. The protocol of the study has been approved by the Human Research Ethics Committee of the Lutheran University of Brazil (ULBRA) and is registered in the National Ministry of Health. This experiment did not affect the medical assistance provided to the patients because it was an analytical review of their medical records. All participants were made aware of the study and provided written consent before the surgery, so that their data, as well as blood level of 25OHD, could be used in research.

The inclusion criteria have been patients aged between 18 and 60 years who had undergone breast augmentation surgery with placement of silicone implants and who underwent a follow-up appointment that was performed at least 2 months after the breast augmentation surgery.

The exclusion criteria have been consumption of supplements with VD or drugs modulating serum 25OHD values, concomitant mastopexy, incomplete medical records, absence of postoperative photos, use of corticosteroids, and previous breast surgery.

**Figure 1:**
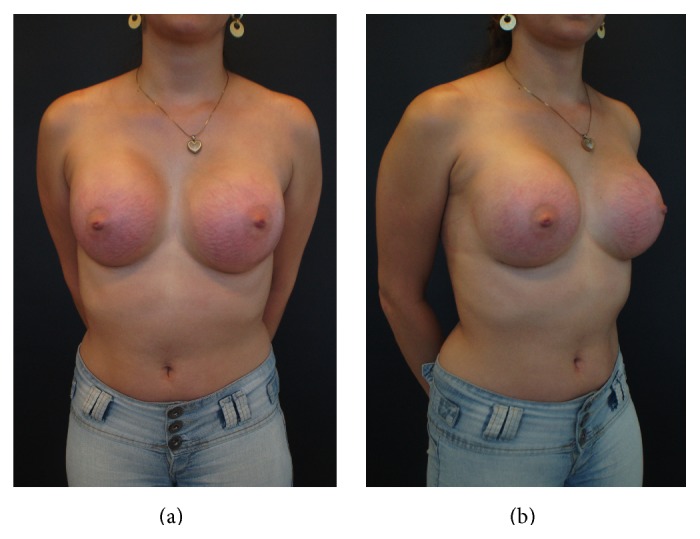
SD 9 weeks after 350 mL breast implants placement in a 29-year-old woman. (a) Frontal view, (b) oblique view.

**Figure 2:**
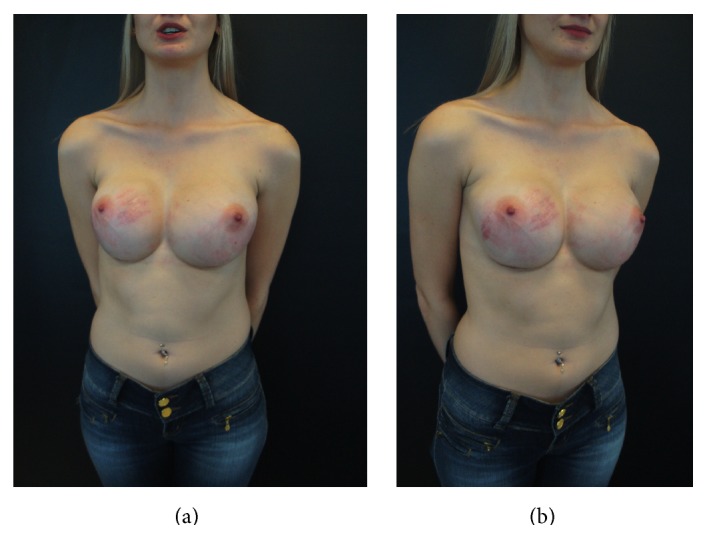
SD 6 weeks after 425 mL breast implants placement in a 24-year-old woman. (a) Frontal view, (b) oblique view.

The criteria to define SD included the presence of cutaneous atrophy with acquired, reddish lesions of linear appearance and with a minimum width of 2 mm. Figures 1 and 2 show these findings. White or silver SD was considered old and was not accounted for by this study.

For each patient diagnosed with SD, four other participants submitted to the same surgery performed by the same surgeon, without the development of SD after breast augmentation surgery, were enrolled as the healthy controls.

Prior to the surgery, blood samples from the patients have been collected by venous puncture. 25OHD values ≥30 ng/mL have been designated as normal, among 20 to 30 ng/mL as inadequate and lower than 20 ng/mL as scarce [13, 14]. 25OHD serum concentration was assayed with an electrochemiluminescence (ECL) method. Data regarding blood level of 25OHD prior to the surgery and SD onset were collected from the electronic records in which information had been entered by the surgeon who performed the procedures.

The obtained data have been evaluated applying Kruskal-Wallis test, one way ANOVA, Student's *t*-test, and Chi-square (linear by linear correlation), as applicable (considering a preset probability of *P* < 0.05). The quantitative data were stated as mean ± standard deviation. The statistical analysis was performed with SPSS (IBM, USA) and Epi Info (CDC, USA) software. The Independent *t*-test has been employed to equate different quantitative variables (including 25OHD serum level, age, body mass index, and implant volume) among SD cases and patients without SD. Farther, the simultaneous effects of the confounding variables like age, implant volume, and body mass index (BMI) have been calculated using simple, multiple, and logistic regression analysis. Kolmogorov-Smirnov test was used to check normality assumptions of distributions. VD values have not reached a normal distribution in the two groups; thus, only logarithmic transformation of VD levels has been compared in both groups.

From an ethical point of view, this study did not affect the medical assistance provided to the patients because it was a simple analytical review of their medical records. Medical confidentiality was assured because the only person responsible for data collection, having access to the identities of the patients, was allowed only to copy information regarding the variables included in this study.

## 3. Results

67 Caucasian women with recognized SD after the surgery entered the study constituting Group I. To form the control group, 268 Caucasian women without recognized SD after the same surgery were enrolled for the study composing Group II.

Comparative analyses between the groups are shown in Table 1. There was no difference in age, BMI, smoking, or implant volume between SD patients and the healthy controls. A statistically significant difference has been detected among the groups regarding the values of 25OHD. In the serum of healthy controls, mean was 27 ng/mL, and SD cases presented a 20 ng/mL serum mean (*P* = 0.01, 95% CI, 1.46–3.86).

Confrontation of serum 25OHD status in Striae Distensae and non-Striae Distensae groups is shown in Table 2. Scarce values of VD have been observed in 38 (56.71%) cases presenting SD and 107 (39.91%) without SD (*P* = 0.002). 16 (23.88%) of SD patients and 56 (20.88%) of the group without SD had inadequate values of VD. Normal values of VD have been detected in 13 (19.41%) SD patients and in 105 (39.21%) patients without SD. Comparing with patients without SD, the chance of having VD values lower than 20 ng/mL amongst cases with SD is 2.38 (95% CI, 1.46–3.86, *P* = 0.0001).

## 4. Discussion

Vitamin D supply is changeable, being sourced from skin synthesis following solar exposure, which is curtailed seasonally in high latitude countries, and from oral intake of natural foodstuffs, fortified foodstuffs, and supplements. Although sunlight exposure is the predominant natural source of vitamin D, the primacy of oral intake over sunlight exposure in both the prevention and correction of vitamin D deficiency has been known for some time. This is apposite given the concerns about sunlight exposure and skin cancer. For these reasons, the Institute of Medicine (IOM) 2011 Report specified dietary reference vitamin D intakes for those with minimal or no sunlight exposure [15]. Individuals with intentional or inadvertent sunlight exposure have lesser dependence on oral sources. The recommended daily allowances specified by IOM in 2011 are between 30% and threefold higher compared to 1997 [16]. Noting the trend for unsubstantiated claims regarding vitamin D, IOM cautioned against exceeding recommended intakes [17].

The IOM gave guidance about the interpretation of the 25OHD result. According to the IOM, 25OHD is a measure of risk: a concentration below 30 nmol/L (12 ng/mL) indicates increased risk of vitamin D deficiency; a concentration of 40 nmol/L (16 ng/mL) corresponds to the estimated average requirement (EAR) satisfying the needs of half the population; a concentration above 50 nmol/L (20 ng/mL) meets the requirements of 97.5% of the population; a concentration above 125 nmol/L (50 ng/mL) indicates risk of harm [13–17]. In our study, we did not found any hypervitaminosis D.

The samples were obtained along all seasons of the year, avoiding a sun exposure bias. Notably, in this study, the sample was obtained from a private clinic, corresponding to a specific social group that does not represent the general population. Therefore, the obtained results and their interpretation must take into account this specificity. The fact that 100% of the sample studied was Caucasian precludes extrapolation of the obtained results to the general population; however, it makes the external validity of this study higher for populations similar to the one used in the study [11].

The physiopathology of SD remains unclear. Excessive skin distention, prolonged exposure to cortisol, and genetics may all have a role. The aetiological mechanisms proposed relate to hormones, and structural alterations to the integument. However, it may involve stretching of the skin, causing lesions in microfibrils, which in younger women are likely to be more fragile and are therefore more susceptible to rupture [11]. Some diseases from the connective tissue, such as congenital contractural arachnodactyly or Marfan syndrome, are also known to be related to SD. Rapid mechanical stretch produced by implant introduction seems to be the main factor leading to SD in the population of our study [12].

Histopathologically, SD shows loss of the supporting material, that is, the collagen and the elastin fibers compared to normal skin, which reduces the thickness and stability of the dermis. Though striae do not present any significant medical issues, they are a cause of cosmetic concern. Many therapeutic modalities have been tried including topical tretinoin, microdermabrasion, radiofrequency, photothermolysis, intense pulsed light, fractional lasers, and ablative and nonablative lasers; but no consistently effective modality is available yet [9, 11].

Hormonal receptor expression is increased under certain conditions suggesting that regions undergoing greater mechanical stretching of the skin may express greater hormonal receptor activity. This fact may influence the metabolism of the extracellular matrix, causing SD formation. Alterations in hormone receptors occur within a well-defined period during the formation of SD. However, the functionality of hormone receptors varies during the different stages of SD development. Estrogen receptors doubled in skin with SD compared with healthy skin. The androgen and glucocorticoid receptors in the SD skin are also increased [11, 18]. Recent evidence supports a positive role for VD in reproductive hormone biosynthesis and ovarian reserve and also a negative role in estrogen receptors; thus, hypovitaminosis D leads to an increase in the estrogen receptors [19, 20]. Probably, this increase in the estrogen receptors observed in hypovitaminosis D can be the explanation to the higher incidence of SD amongst women with lower levels of VD.

We choose to conduct a case-referent study because it is good for studying rare conditions, needs less time to conduct the study because the condition has already occurred, and is useful as an initial study to establish an association. Knowing its disadvantages, we avoid recall bias doing patients' electronic records review to find a suitable control group four times bigger than the SD group. We have used our own previously published work [11], as a basis for this paper; the novel contribution beyond those of the previous paper is the levels of VD study. In our prior study, we did not mention vitamin D along the manuscript. Further studies with VD measurement must be performed in other groups with increased risk to develop SD (during pregnancy, hormonal growth spurts, obesity, or rapid weight gain).

## 5. Conclusion

Lower serum levels of VD are linked to a higher occurrence of SD. Further studies are needed to elucidate a potentially different impact of hypovitaminosis D on SD.

## Figures and Tables

**Table 1:** Comparison of cases in Striae Distensae and non-Striae Distensae groups.

Parameter	Striae Distensae group (*n* = 67)	Healthy controls (*n* = 268)	*P* value
25OHD (ng/mL)	20 (2.9)	27 (2.2)	0.01
Age (years)	25 (4.2)	26 (4.1)	0.51
BMI (kg/m^2^)	22.24 (1.59)	21.96 (1.52)	0.39
Current smoker (%)	8.95	7.46	0.27
Implant volume (cc)	327.28 (64.1)	324.91 (61.9)	0.61

**Table 2:** Confrontation of serum 25OHD status in Striae Distensae and non-Striae Distensae groups.

Vitamin D status	Striae Distensae group (*n* = 67)	Healthy controls (*n* = 268)	*P* value
Scarce (<20 ng/mL)	38 (56.71%)	107 (39.91%)	0.002
Inadequate (20–30 ng/mL)	16 (23.88%)	56 (20.88%)	
Normal (>30 ng/mL)	13 (19.41%)	105 (39.21%)	
